# Prediction of recurrence-free survival using a protein expression-based risk classifier for head and neck cancer

**DOI:** 10.1038/oncsis.2015.7

**Published:** 2015-04-20

**Authors:** S S Chauhan, J Kaur, M Kumar, A Matta, G Srivastava, A Alyass, J Assi, I Leong, C MacMillan, I Witterick, T J Colgan, N K Shukla, A Thakar, M C Sharma, K W M Siu, P G Walfish, R Ralhan

**Affiliations:** 1 Department of Biochemistry, All India Institute of Medical Sciences, New Delhi, India; 2 Alex and Simona Shnaider Laboratory in Molecular Oncology, Mount Sinai Hospital, Toronto, Ontario, Canada; 3 Department of Clinical Epidemiology and Biostatistics, McMaster University, Hamilton, Ontario, Canada; 4 Department of Pathology and Laboratory Medicine, Mount Sinai Hospital, Joseph & Wolf Lebovic Health Complex, Toronto, Ontario, Canada; 5 Department of Oral Pathology and Oral Medicine, Faculty of Dentistry, University of Toronto, Toronto, Ontario, Canada; 6 Department of Otolaryngology – Head and Neck Surgery, Joseph and Mildred Sonshine Family Centre for Head and Neck Diseases, Mount Sinai Hospital, Toronto, Ontario, Canada; 7 Department of Otolaryngology – Head and Neck Surgery, University of Toronto, Toronto, Ontario, Canada; 8 Department of Surgery, Dr. B.R.A. Institute Rotary Cancer Hospital, All India Institute of Medical Sciences, New Delhi, India; 9 Department of Otorhinolaryngology, All India Institute of Medical Sciences, New Delhi, India; 10 Department of Pathology, All India Institute of Medical Sciences, New Delhi, India; 11 Department of Chemistry and Biochemistry, University of Windsor, Windsor, Ontario, Canada; 12 Endocrine Division, Department of Medicine, Mount Sinai Hospital and University of Toronto, Toronto, Ontario, Canada

## Abstract

Loco-regional recurrence in 50% of oral squamous cell carcinoma (OSCC) patients poses major challenge for oncologists. Lack of biomarkers that can predict disease aggressiveness and recurrence risk makes the scenario more dismal. On the basis of our earlier global proteomic analyses we identified five differentially expressed proteins in OSCC. This study aimed to develop protein biomarkers-based prognostic risk prediction model for OSCC. Sub-cellular expression of five proteins, S100A7, heterogeneous nuclear ribonucleoproteinK (hnRNPK), prothymosin α (PTMA), 14-3-3ζ and 14-3-3σ was analyzed by immunohistochemistry in test set (282 Indian OSCCs and 209 normal tissues), correlated with clinic–pathological parameters and clinical outcome over 12 years to develop a risk model for prediction of recurrence-free survival. This risk classifier was externally validated in 135 Canadian OSCC and 96 normal tissues. Biomarker signature score based on PTMA, S100A7 and hnRNPK was associated with recurrence free survival of OSCC patients (hazard ratio=1.11; 95% confidence interval 1.08, 1.13, *P*<0.001, optimism-corrected c-statistic=0.69) independent of clinical parameters. Biomarker signature score stratified OSCC patients into high- and low-risk groups with significant difference for disease recurrence. The high-risk group had median survival 14 months, and 3-year survival rate of 30%, whereas low-risk group survival probability did not reach 50%, and had 3-year survival rate of 71%. As a powerful predictor of 3-year recurrence-free survival in OSCC patients, the newly developed biomarkers panel risk classifier will facilitate patient counseling for personalized treatment.

## Introduction

Head and neck squamous cell carcinoma (HNSCC) with over 600 000 new cases diagnosed annually persists as a formidable clinical challenge and ranks as the sixth leading cause of cancer deaths worldwide.^1, 2^ HNSCC shows heterogeneous pathologic and clinical features with diverse outcome; the clinical and histologic appearance of the oral mucosa may not fully disclose the damage at molecular level.^3, 4^ The survival rate for early diagnosed HNSCC patients is about 82.4% within first 5 years; whereas for those in late stages is 34.9% (www.seer.cancer.gov). HNSCC patients often have tumor recurrence at the same site, or develop second primary tumors, frequently attributed to field cancerization.^5^ Oral squamous cell carcinomas (OSCCs) comprise a large proportion of HNSCC. The lack of clinically proven biomarkers limits therapeutic decisions to be solely based on clinicopathological parameters; tumors with similar clinical features can differ in disease outcome.^6^ There is urgent need for prognostic biomarkers for the stratification of patients with high risk of disease recurrence for more rigorous management.

High-resolution genomic and proteomic profiling is being used to develop panels of biomarkers to predict therapeutic response or disease prognosis.^7, 8^ Several studies reported genome-wide profiling of HNSCC.^9, 10, 11, 12, 13, 14, 15, 16^ Gene expression signatures and microRNAs correlating with poor prognosis have been identified.^16, 17, 18, 19, 20, 21, 22, 23, 24, 25, 26, 27^ Proteomic studies demonstrated alterations in protein profiles in OSCC.^28, 29^ Novel biomarkers that are associated with biologic behavior of OSCC are needed to improve the management and personalization of treatment. We reported quantitative tissue proteomics analyses of oral premalignant lesions and OSCCs using isobaric mass tags (iTRAQ) and mass spectrometry based panel of five candidate biomarkers: S100A7, 14-3-3ζ, 14-3-3σ, prothymosin-α (PTMA) and heterogeneous nuclear ribonucleoproteinK (hnRNPK).^28, 29, 30^ Subsequently, others reported proteomic markers for OSCC.^6, 31, 32, 33, 34, 35^ Yet, none of these markers have been validated for clinical use.

The objective of this study was to evaluate this panel of prognostic markers S100A7, 14-3-3ζ, 14-3-3σ, PTMA and hnRNPK for OSCC patients. The rationale for selecting these proteins was based on their biological functions as well as distinctive and independent associations with oral cancer development and progression in our earlier individual biomarker studies.^30, 36, 37, 38^ These proteins are deregulated in molecular pathways that have pivotal role in acquisition of aggressive features (changes in cell–cell adhesion and interactions with extracellular matrix, cell proliferation, cell signaling and apoptosis).^36, 37, 38^ Here in we conducted retrospective studies in large cohort of OSCCs comprising of two patient populations, Indian and Canadian, to analyze the correlations of alterations in sub-cellular expression of these proteins with clinical and pathological parameters and follow-up for disease free survival. Biomarkers- and clinical parameters-based overall signature score was used to develop a protein expression-based risk prediction model for recurrence-free survival of OSCC patients, as a step forward towards establishing their clinical applicability that is likely to have implications for personalized therapy.

## Results

### Validation of overexpression of the panel of five proteins in OSCCs in comparison with normal tissues

**Figure 1:**
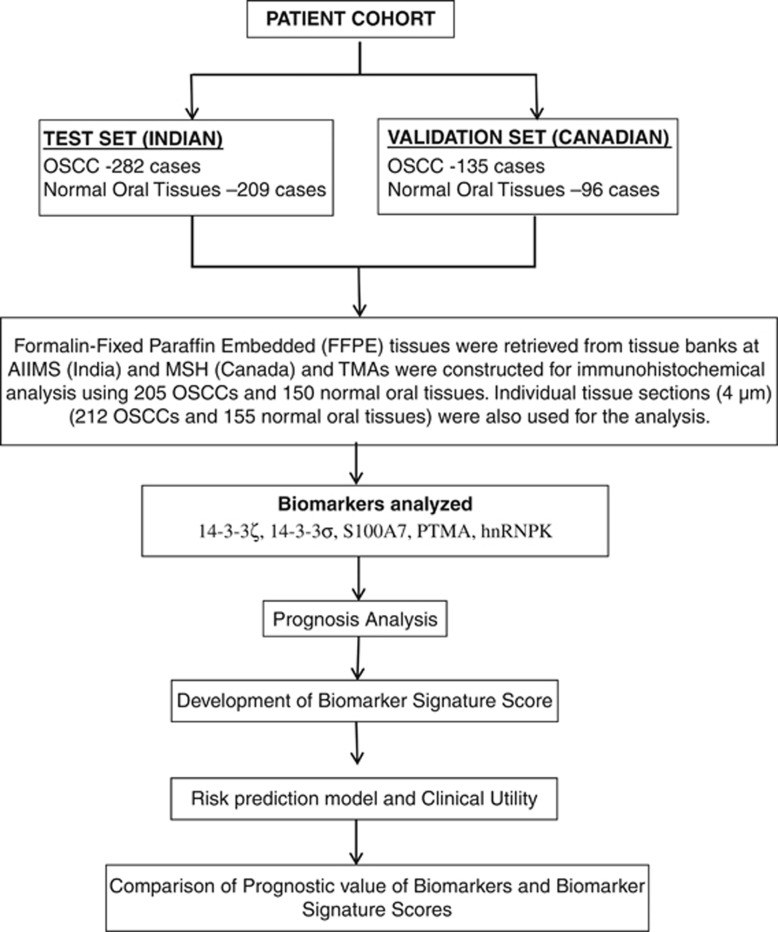
Schematic design of the study.

The study design is outlined in Figure 1. The demographical and clinical parameters for the two sets (test and validation) are outlined in Table 1. Immunostaining for five proteins was performed in OSCCs (test set *n*=282 and validation set *n*=135) and normal oral tissues (test set *n*=209 and validation set *n*=96) and scored. The analyses for variations in expression levels of the five proteins in normal oral tissues and in OSCCs are summarized in Supplementary Tables S1 and S2 for the test and validation sets, respectively. The correlations of protein expressions with patients' demographic characteristics (age and gender) as well as clinical and pathological parameters (tumor site, histopathological grade, tumor stage, nodal status and clinical stage) are given in Supplementary Tables S1 and S2. Our data validated significant overexpression of S100A7, PTMA, hnRNPK, 14-3-3ζ and 14-3-3σ in cytoplasm and/or nucleus of OSCC as compared with normal and their association with clinical and pathological parameters in test set (*P*<0.001, Supplementary Table S1). These results were fairly replicated in the validation set (Supplementary Table S2). The distributions of biomarker scores for OSCCs were found to be fairly consistent in the test and validation sets suggesting a stable replication that capture the overall variability in proteins expressions (Supplementary Figure S1).

### Assessment of biomarkers' prognostic value as a panel

A panel of biomarkers comprising of nuclear S100A7, cytoplasmic hnRNPK, nuclear PTMA and cytoplasmic PTMA were observed to be predictive for time of recurrence (Supplementary Table S3). Nuclear S100A7, cytoplasmic hnRNPK and nuclear PTMA, were associated with poor prognosis, whereas cytoplasmic PTMA was associated with good prognosis. The prognostic value of this panel was internally and externally validated and multivariate regression estimates were fairly similar (Supplementary Table S4). Hence, these three biomarkers, nuclear S100A7, cytoplasmic hnRNPK and nuclear PTMA, hold significant prognostic values independent of each other, and more importantly, improve disease prognosis assessment as a panel. Clinical parameters including differentiation and nodal status do show a prognostic value when analyzed alone and/or together which confirms the quality of our data (Table 2).

### Development of biomarker signature score

**Figure 2:**
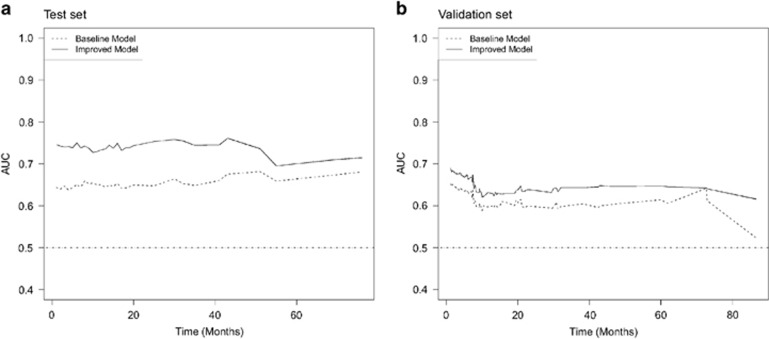
(**a**) Test set. Time-dependent AUC plot of biomarkers improvement upon clinical parameters. The baseline model (dashed line) is fitted using age, gender, site, histology grade, nodal status, clinical stage and tumor stage. The biomarkers considered were nuclear S100A7, cytoplasmic hnRNPK, nuclear PTMA and cytoplasmic PTMA. The improved baseline model (solid line) was fitted using clinical parameters extended by the biomarker signature score. (**b**). Validation set. Time-dependent AUC plot of biomarkers improvement upon clinical parameters. The baseline model (dashed line) is fitted using age, gender, site, histology grade, nodal status, clinical stage and tumor stage. The biomarkers considered were nuclear S100A7, cytoplasmic hnRNPK, nuclear PTMA and cytoplasmic PTMA. The improved baseline model (solid line) was fitted using clinical parameters extended by the biomarker signature score.

Biomarkers signature score was calculated as a linear combination of nuclear PTMA, cytoplasmic PTMA, nuclear S100A7 and cytoplasmic hnRNPK, with regression estimates as weights (score=1.4 × nuclear S100A7+2.1 × nuclear PTMA−1.9 × cytoplasmic PTMA; Tables 2 and 3). Biomarkers signature score was associated with time of recurrence (HR (hazard ratio)=1.11 (95% CI (confidence interval)=1.08, 1.13); *P*<0.001), and achieved a discriminatory c-statistic value of 0.69. The biomarkers signature score was also found to hold a prognostic value adjusted for those clinical parameters, and does improve upon them. The reference baseline model achieved a discriminatory c-statistic of 0.60. Adding the clinical parameters only marginally improved the discriminatory value to 0.70, suggesting a clinical value of these biomarkers signature score. Overall, the prognostic value of this biomarkers signature score adds improvements to the classical clinical parameters for assessing prognosis of OSCC patients. The time-dependent area under the curve (AUC) plot of the baseline and improved baseline models confirmed that biomarkers together with clinical parameters (age, gender, histopathological grade, nodal status, tumor stage and clinical stage) hold better overall discriminatory ability throughout time compared with the use of clinical parameters alone (Figure 2). Several models including the interaction terms of (1) nodal status with biomarker signature score, (2) tumor stage with biomarker signature score, (3) clinical stage with biomarker signature score and (4) histology grade with biomarker signature score, were further explored in the Test and Validation sets. No significant and stable interactions were observed, and this suggests biomarker signature score is independent of clinical parameters (Supplementary Table S5).

### Clinical utility of biomarker signature score

**Figure 3:**
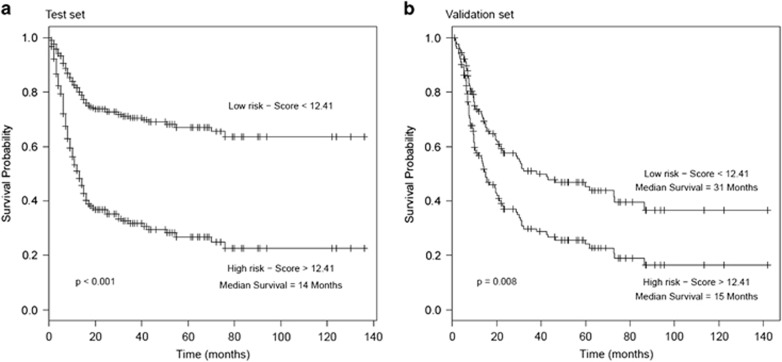
(**a**) Kaplan–Meier survival analysis of OSCC patients stratified into high- and low-risk groups in test set. (**b**) Kaplan–Meier survival analysis of OSCC patients stratified into high- and low-risk groups in validation set.

A cut off was derived from the test set as the median risk score to stratify subjects into high- and low-risk groups of recurrence (score=12.41). A HR estimate for the prognostic value of stratification via biomarker signature score into high- and low-risk groups was found to be clinically and statistically significant (training set: HR=3.30 (95% CI=2.23, 4.86) *P*<0.001; validation set: 1.79 (95% CI=1.15, 2.79), *P*=0.009). Kaplan–Meier survival analyses show that two risk groups in the test and validations sets have significantly different survival times (log-rank test: *P*<0.001, and *P*=0.008; Figure 3). The high-risk group had a median survival time of 14 and 15 months in the test and validation sets, respectively. The low-risk group in comparison had a median survival time of 31 months in the validation set and did not reach a survival probability <50% in the test set. The 3-year disease-free survival rate for patients in the high-risk group was 30% (95% CI=22%, 41%) in comparison to 71% (95% CI=0.64%, 80%) in the low-risk group. These results were fairly replicated in the validation set with survival rates of 32% (95% CI=22%, 48%) and 50% (95% CI=38%, 63%) for the high- and low-risk groups, respectively. The replication of these results in the test and validation sets verifies the clinical utility of biomarkers risk score in different patient populations. The clinical utility of biomarker signature score cut off value of 12.41 was also assessed using its ability to correctly identify subjects at high- and low-risk of recurrence/death within 5 years post surgery. Using a cut off value of 12.41, 86 and 83% of patients within the high-risk groups had recurrence/death 5 years, within the test and validation sets, respectively.

## Discussion

Our study uniquely based on sub-cellular compartment analysis of expression of a panel of five proteins, taking into account the percentage positivity and intensity of immunostaining, for correlation with clinical outcome, gave a comprehensive insight into their clinical relevance on disease outcome. The association of three of these five biomarkers analyzed with disease prognosis was validated in these independent cohorts of OSCCs comprising of Canadian and Indian patients. Importantly, we identified and demonstrated that this panel of three biomarkers constituted the prognostic molecular signature for OSCC patients. Our panel of biomarkers predicted disease recurrence more effectively as compared with individual biomarkers. These findings demonstrated the strong predictive power of our panel of biomarkers for OSCC patients.

Multivariable Cox regression analyses and time-dependent AUC plots showed that our panel of biomarkers not only has a better discriminatory value, but adds upon clinical parameters including histology grade, nodal status, tumor stage and clinical stage. Hence, we have confirmed the clinical usefulness of this promising panel of biomarkers by their ability to add unique prognostic information to the clinical predictors—histological grade, nodal status and tumor stage. Notably, a risk stratification based on biomarkers signature score identified participants at high- and low-risk groups for recurrence. Kaplan–Meier analyses determined a 3-year survival rate of 30% and 71% for participants in the high- and low-risk groups, respectively.

However, our study is not devoid of limitations. First, the end point of this study was disease recurrence. Although this is a surrogate end point for clinical progression, not all patients with recurrence will progress to distant metastases and/or cancer-related death. Unfortunately, the natural history of oral cancer limits the availability of more definitive end points. Secondly, the test and validation sets were not fully segregated up till the final evaluation. The biomarkers that showed prognostic potential in the test set but were not observed to be associated with disease prognosis within the validation set were excluded from the biomarkers signature score due to stability issues. Despite these limitations, we were able to demonstrate a significant relationship between these biomarkers of interest, and also showed better accuracy of the biomarkers in identifying OSCC patients at higher risk of recurrence of the disease. Further, the clinical implementation of our panel of biomarker signature score-based test uses the technique of immunohistochemical analysis that is routinely performed in most pathological laboratories and thus easy to translate from bench to clinic. Another major advantage of our proteins-based test panel is its cost effectiveness which is generally less than the cost of gene signature-based tests.

The internal and external validations demonstrate the stability of our biomarker signature score utility in clinical settings based on Canadian and Indian OSCC patients. Our study assumes considerable importance because these biomarkers largely hold their significance in the Canadian and Indian data sets analyzed separately as well. Further, biomarkers signature score retain their significant association with disease prognosis in the two patient populations. It is well known that the etiology and risk factors associated with oral cancer in the North American and South Asian populations are considerably different. Yet our protein expression-based risk classifier model shows promise in Canadian and Indian OSCC patients, suggesting it is likely to have widespread clinical utility for prediction of recurrence free survival of OSCC patients. These findings set the stage for independent multicentric prospective studies to assess if this risk classifier could help to predict recurrence-free survival that can be used to guide clinical management of OSCC in future. In comparison most gene expression signatures in head and neck cancer are discovery phase reports and their association with clinical outcome await validation.^22, 39, 40, 41^

In conclusion, integrated analysis of expression of the panel of three proteins on two important patients' populations allowed us to validate the robustness of our biomarker panel in stratification of OSCC patients at high or low risk of disease recurrence. This risk classifier has the potential to identify the high-risk patients for more rigorous personalized treatment, whereas the low-risk patients can be kept under active surveillance, but spared from the harmful side effects of toxic therapy as well as reduce the burden on health care providers. The findings of our study set the foundations for translation of this panel of protein markers for OSCC patients and establish their clinical relevance for larger worldwide application in future studies.

## Patients and methods

### Patient selection

This retrospective study was approved by Research Ethics Board (REB) of All India Institute of Medical Sciences (AIIMS), New Delhi, India, and Mount Sinai Hospital (MSH), Toronto, Canada, prior to its commencement. The Reporting Recommendations for Tumor Marker prognostic Studies (REMARK) criteria were followed throughout this study.^42^ *I*nclusion criteria: patients with histopathological evidence of OSCC confirmed by a pathologist and known clinical outcome. Exclusion criteria: patients diagnosed with cancer of the oral cavity but with no available follow-up data. Patient demographic, clinical and pathological data were recorded in a predesigned Performa as described by us earlier.^38^ The information documented included clinical TNM staging (based on the Union International Center le Cancer TNM classification of malignant tumors 1998), site of the lesion, histopathological grade, age, gender and treatment. Following the above inclusion and exclusion criteria, archived formalin-fixed paraffin-embedded (FFPE) tissue specimens of OSCC patients (*n*=417, median age: 53 years; range: 19–92 years) undergoing curative surgery during the period 2000–2007 were inducted into this study. The OSCC patients cohort comprised of 282 cases (median age: 49 years; range: 19–85 years) from AIIMS and 135 cases (median age 63 years; range 21–92 years) from MSH. The normal group comprised of 305 histologically normal oral tissues confirmed by hematoxylin and eosin stain staining. Of these, 209 paired normal tissues were from AIIMS and 96 tissues were from MSH. All OSCC patients were treated as per the National Comprehensive Cancer Network (NCCN) guide lines for head and neck cancers (www.nccn.org). As per the hospital protocol, OSCC patients with T_1_ and T_2_ tumors were treated with radical surgery; majority of patients with T_3_ and T_4_ disease were treated with radical surgery followed by postoperative radical radiotherapy.^38^

### Follow-up study

All OSCC patients were followed in the cancer follow-up clinics for a maximum period of 136 months (mean 23.5 months, median 14 months), and 142 months (mean 30 months, median 15.5 months) in the AIIMS and MSH centers, respectively. Recurrence or death was observed in 122 of 282 (43.3%), and in 80 of 135 (59.3%) patients in the AIIMS and MHS centers, respectively. The patients revisited clinic regularly and time to recurrence was recorded. If a patient died, the survival time was censored at the time of death; the medical history, clinical examination and radiological evaluation were used to determine whether the death had resulted from recurrent cancer (relapsing patients) or from any other causes. Disease-free survivors were defined as patients free from clinical and radiological evidence of local, regional or distant relapse at the time of last follow-up. Follow-up period was defined as the interval from the time when patient underwent first surgery to recurrence of cancer or death (for uncensored observations) or no recurrence at last consultation (for censored observations).

### Tissue microarrays (TMAs) construction and immunohistochemistry

The histopathologic diagnoses were reconfirmed by oral pathologists. Tissue sections comprising of over 70% epithelial cells (cancer / normal) were selected for immunohistochemistry. Of the 417 OSCCs and 305 normal tissue blocks, 205 OSCCs and 150 normals were used for construction of TMAs, whereas the remaining were used as individual sections for immunostaining. Consecutive 4 μm sections were cut from the recipient block and used for immunohistochemical staining for above mentioned five proteins.^36^ The TMA blocks were constructed by relocating small cylindrical tissue cores (two cores per tissue block representing the cancer sections) from individual donor blocks and placing them in a recipient block with defined array coordinates. Arrays were constructed from FFPE tissues by the removal of 0.6 mm diameter tissue cores from donor blocks. A total of two morphologically representative areas of interest from each donor block were identified under the microscope by the pathologists using a stained hematoxylin and eosin section as a guide. Using a precise spacing pattern on manual TMA instrument, 150–200 cores could be transferred to the recipient paraffin block in a grid like fashion, retaining a link to the original block and its pathology.^36^

TMAs/tissue sections were immunostained using Vectastain Elite ABC kit (PK-6100) rapid protocol (Vectastain Laboratories, Burlingame, CA, USA). After antigen retrieval, slides were immunostained with respective mouse monoclonal antibodies; anti-S100A7 (1:500 dilution; sc-52948, Santa Cruz Biotechnology, Santa Cruz, CA, USA); anti-PTMA (1:3500; LS-B2322, Lifespan Biosciences, Seattle, WA, USA); anti-hnRNPK (1:5000; ab23644, Abcam, Cambridge, MA, USA); anti-14-3-3σ (1:2500; ab14116-50, Abcam); 14-3-3ζ (1:100; IMG-6664A, Imgenex, San Diego, CA, USA) as described.^30, 37, 38, 43^ The specificities of these antibodies for use in immunohistochemical assays for these proteins had been confirmed in our earlier studies.^30, 36, 37, 38^ The sections were evaluated by light microscopic examination. Images were captured using the Visiopharm Integrator System (Horsholm, Denmark). Tissue sections from cancers known to over-express these proteins were used as a positive control and isotype specific mouse IgG was used as negative control in each batch of immunohistochemistry.

### Selection of cut off scores

Immunopositive staining was evaluated in each core on TMA and five areas of the tissue sections as described by us earlier.^9, 18, 22, 23, 43^ Sections were scored as positive if epithelial cells showed immunopositivity in cytoplasm, and/or nucleus observed by the evaluators who were blinded to clinical outcome. These sections were scored as follows: 0, <10% cells; 1, 11–30% cells; 2, 31–50% cells; 3, 51–70% cells; and 4, >70% cells showed immunoreactivity. Sections were also scored semi-quantitatively on the basis of intensity as follows: 0, none; 1, mild; 2, moderate; and 3, intense.

### Statistical analysis

The relationships between these proteins and patients characteristics were compared using Kruskal–Wallis rank sum tests. The distribution of biomarker scores in the test and validation sets were assessed using histograms. Cox regression analyses were used to assess the prognostic value of biomarkers and clinical parameters in the test and validation sets. Stepwise variable selection was used in the test set to acquire a panel of biomarkers in which a signature score was derived. The response was the time-to-event of recurrence, while the predictors are ordinal biomarker scores. A signature score is the linear combination of biomarker expressions using regression estimates as weights. Optimism-corrected Harrell's c-statistic was used to summarize the overall discriminatory value of biomarkers signature score.^44^ Cox regression analyses were internally and externally validated. Internal validations and corrections for optimism were done using the bootstrap approach with 9999 replications via resampling with replacement.^44^ Improvements by biomarkers signature score upon clinical parameters were assessed by multivariable Cox regression analyses and time-dependent AUC plots. Interactions tests between biomarkers signature score and clinical parameters were also performed. Cox proportional hazards assumption was ensured via chi-squared test for goodness of fit on Schoenfeld residuals.^45^ A median risk score value derived from test set was used to classify subjects into high- and low-risk groups of recurrence and further verified in the validation set. Kaplan–Meier survival curves of were used to assess survival time of participants in the high- and low-risk groups. All statistical analyses were carried out using R version 3.01 (http://www.r-project.org/). Cox proportional hazard models were fitted using *rms* package in R.^46^ Time-dependent AUC plots were done using *riskset ROC* R package.^47^

## Figures and Tables

**Table 1:** Immunohistochemical analysis of five biomarkers in normal oral tissues and OSCCs within test and validation sets

*Clinical features*	*Test set*	*Validation set*
Normal	209	96
Cancer	282	135
Age (years)^a^	49 (38,60)	63 (53,74)
		
*Gender*		
Female	70 (33%)	52 (39%)
Male	212 (67%)	83 (61%)
		
*Site*		
Alveolus	39 (14%)	2 (1.5%)
BM	108 (38%)	14 (10%)
Mandible	4 (1%)	8 (6%)
Lip	6 (2%)	1 (1.5%)
Palate	8 (3%)	2 (1%)
RMT	10 (4%)	0 (0%)
Tongue	98 (35%)	108 (80%)
Others	9 (3%)	0 (0%)
		
*HP grade*		
WDSCC	166 (59%)	33 (25%)
MDSCC	106 (37%)	87 (64%)
PDSCC	10 (4%)	15 (11%)
		
*T stage*		
T_1_ and T_2_	77 (27%)	102 (75%)
T_3_ and T_4_	205 (73%)	33 (25%)
		
*Node*		
N^−^	99 (35%)	76 (56%)
N^+^	183 (65%)	59 (44%)
		
*Clinical stage*		
I and II	33 (12%)	62 (46%)
III and IV	249 (88%)	73 (54%)
Biomarker risk score^a^	12 (6.3, 17)	11 (9.0, 15)

Abbreviations: BM, buccal mucosa; HP grade, histopathological grade; RMT, retro molar trigone; T stage, tumor stage.

a Median (25th and 75th percentiles).

**Table 2:** Univariable Cox regression analysis of clinical parameters and biomarkers risk score

*Predictors*	*Test set (*n=*282, events=122)*			*Internal validation 9999 bootstrap samples*			*External validation (*n=*135, events=80)*		
	*HR (95% CI)*	P*-value*	C^a^	*HR (95% CI)*	P*-value*	C^a^	*HR (95% CI)*	P*-value*	C^a^
Age	1.00 (0.98, 1.01)	0.84	0.51	1.00 (0.98, 1.01)	0.84	0.49^b^	0.99 (0.97, 1.01)	0.37	0.54
Gender	1.28 (0.84, 1.97)	0.25	0.52	1.29 (0.85, 2.04)	0.26	0.52^b^	1.00 (0.64, 1.57)	0.99	0.50
Histology grade	1.58 (1.19, 2.11)	0.002	0.56	1.59 (1.2, 2.11)	0.001	0.56^b^	1.17 (0.80, 1.71)	0.41	0.54
Nodal status	2.61 (1.68, 4.07)	<0.001	0.59	2.64 (1.71, 4.35)	<0.001	0.59^b^	1.76 (1.13, 2.73)	0.01	0.60
Tumor stage	1.73 (1.12, 2.67)	0.01	0.55	1.75 (1.14, 2.82)	0.02	0.54^b^	1.48 (0.91, 2.41)	0.12	0.56
Clinical stage	1.77 (0.95, 3.3)	0.07	0.53	1.79 (1, 3.91)	0.10	0.53^b^	1.40 (0.90, 2.19)	0.14	0.57
Biomarker signature score	1.11 (1.08, 1.13)	< 0.001	0.69	1.11 (1.08, 1.14)	<0.001	0.69^b^	1.06 (1.01, 1.12)	0.02	0.59

Abbreviations: CI, confidence interval; HR, hazard ratio.

a c-Statistics.

b Optimism-corrected index.

**Table 3:** Multivariable Cox regression analysis for biomarkers signature score improvements upon clinical parameters

*Predictors*	*Test set (*n=*282, events=122)*		*Internal validation 9999 bootstrap samples*		*External validation (*n=*135, events=80)*	
	*HR (95% CI)*	P*-value*	*HR (95% CI)*	P*-value*	*HR (95% CI)*	P*-value*
*Baseline model based on demographics and clinical parameters*						
Age	1.01 (0.99, 1.02)	0.46	1.01 (0.99, 1.02)	0.48	0.99 (0.97, 1.01)	0.26
Gender	1.15 (0.74, 1.77)	0.53	1.14 (0.75, 1.83)	0.54	1.21 (0.75, 1.96)	0.43
Histopathological grade	1.50 (1.11, 2.02)	0.007	1.50 (1.12, 2.00)	0.006	1.14 (0.76, 1.71)	0.53
Nodal status	2.56 (1.56, 4.21)	<0.001	2.61 (1.57, 4.71)	<0.001	4.99 (1.96, 12.7)	<0.001
Tumor stage	1.47 (0.90, 2.41)	0.13	1.50 (0.92, 2.62)	0.15	2.97 (1.49, 5.91)	0.002
Clinical stage	0.77 (0.36, 1.66)	0.51	0.77 (0.33, 1.90)	0.56	0.23 (0.08, 0.70)	0.009
Discriminatory value	c-statistics=0.62		c-statistics=0.60^a^		c-statistics=0.61^a^	
						
*Improved model using biomarker signature score*						
Biomarker signature score	1.10 (1.07, 1.13)	<0.001	1.10 (1.07, 1.13)	<0.001	1.08 (1.02, 1.15)	0.009
Age	1.00 (0.99, 1.01)	0.97	1.00 (0.99, 1.01)	0.97	0.99 (0.97, 1.01)	0.19
Gender	1.12 (0.73, 1.74)	0.60	1.11 (0.73, 1.75)	0.60	1.29 (0.80, 2.09)	0.29
Histopathological grade	1.33 (0.98, 1.79)	0.06	1.32 (1.00, 1.77)	0.05	1.11 (0.74, 1.67)	0.61
Nodal status	2.29 (1.41, 3.71)	<0.001	2.37 (1.45, 4.29)	0.03	4.77 (1.87, 12.2)	0.001
Tumor stage	1.52 (0.92, 2.50)	0.10	1.54 (0.91, 2.72)	0.14	3.05 (1.53, 6.06)	0.001
Clinical stage	0.79 (0.37, 1.68)	0.53	0.76 (0.30, 1.91)	0.61	0.27 (0.09, 0.80)	0.02
Discriminatory value	c-statistics=0.71		c-statistics=0.70^a^		c-statistics=0.64^a^	

Abbreviations: CI, confidence interval; HR, hazard ratio.

a Optimism-corrected index.

## Supplementary Material

Supplementary Information

Supplementary Figure S1A

Supplementary Figure S1B

Supplementary Figure S1C

Supplementary Figure S1D

Supplementary Figure S1E
