# Supplementary material for: Prediction of recurrence-free survival using a protein expression-based risk classifier for head and neck cancer
Source: Oncogenesis. 2015 Apr 20;4(4):e147–. doi: 10.1038/oncsis.2015.7 (PMC4491610; doi:10.1038/oncsis.2015.7)
Supplement: Supplementary Information [file oncsis20157x1.doc]

Table S1. Immunohistochemical analysis of five biomarkers in normal oral tissues and OSCCs within Test set

| **Clinical features** |  | **hnRNPK** | | **S100A7** | | **PTMA** | | **14-3-3σ** | | **14-3-3ζ** | |
| --- | --- | --- | --- | --- | --- | --- | --- | --- | --- | --- | --- |
|  | **N** | **Cytoplasmic** | **Nuclear** | **Cytoplasmic** | **Nuclear** | **Cytoplasmic** | **Nuclear** | **Cytoplasmic** | **Nuclear** | **Cytoplasmic** | **Nuclear** |
|  |  | **Mean (SD)** | **Mean (SD)** | **Mean (SD)** | **Mean (SD)** | **Mean (SD)** | **Mean(SD)** | **Mean (SD)** | **Mean (SD)** | **Mean (SD)** | **Mean (SD)** |
| **Normal** | 209 | 0.11 (0.70) | 2.93 (2.52) | 1.39 (2.20) | 0.53 (1.61) | 0.66 (1.52) | 1.16 (1.98) | 4.41 (2.25) | 1.44 (2.27) | 4.15 (2.00) | 1.16 (1.87) |
| **Cancer** | 282 | 2.31 (2.56) ^a^ | 4.71 (2.46) ^a^ | 3.54 (2.73) ^a^ | 2.84 (2.66) ^a^ | 1.57 (2.22) ^a^ | 3.69 (2.64) ^a^ | 5.15 (1.77) ^a^ | 2.62 (2.52) ^a^ | 5.76 (1.61) ^a^ | 2.98 (2.72) ^a^ |
| **Age (years)** |  |  |  |  |  |  |  |  |  |  |  |
| < 49 | 140 | 2.24 (2.58) | 4.49 (2.59) | 3.47 (2.77) | 2.81 (2.67) | 1.41 (2.14) | 3.45 (2.65) | 5.17 (1.82) | 2.43 (2.50) | 5.74 (1.57) | 3.00 (2.69) |
| ≥ 49 | 142 | 2.38 (2.55) | 4.94 (2.32) | 3.62 (2.69) | 2.88 (2.65) | 1.74 (2.30) | 3.93 (2.62) | 5.14 (1.72) | 2.80 (2.53) | 5.78 (1.65) | 2.96 (2.76) |
| **Gender** |  |  |  |  |  |  |  |  |  |  |  |
| Female | 70 | 2.46 (2.65) | 4.83 (2.58) | 3.50 (2.63) | 2.76 (2.52) | 1.68 (2.30) | 3.66 (2.59) | 5.33 (1.61) | 2.84 (2.60) | 6.09 (1.11) | 3.22 (2.85) |
| Male | 212 | 2.26 (2.54) | 4.68 (2.43) | 3.56 (2.76) | 2.87 (2.71) | 1.54 (2.20) | 3.70 (2.66) | 5.10 (1.82) | 2.54 (2.49) | 5.65 (1.73) ^b^ | 2.90 (2.68) |
| **Site** |  |  |  |  |  |  |  |  |  |  |  |
| Alveolus | 39 | 3.29 (2.68) | 4.65 (2.64) | 4.28 (2.65) | 3.11 (2.76) | 1.99 (2.58) | 4.08 (2.49) | 5.69 (1.15) | 3.48 (2.54) | 6.15 (1.14) | 3.62 (2.75) |
| BM | 108 | 2.04 (2.54) | 4.55 (2.43) | 3.20 (2.75) | 2.66 (2.67) | 1.47 (2.22) | 3.34 (2.75) | 4.95 (2.05) | 2.34 (2.44) | 5.73 (1.69) | 3.44 (2.71) |
| Mandible | 4 | 4.88 (1.44) | 6.00 (0.00) | 3.00 (3.56) | 2.00 (2.83) | 3.98 (1.36) | 5.70 (0.48) | 5.90 (0.12) | 4.00 (1.41) | 6.25 (0.41) | 1.25 (1.50) |
| Lip | 6 | 1.60 (2.48) | 4.33 (2.42) | 3.83 (3.06) | 3.50 (3.02) | 1.47 (2.29) | 4.17 (2.71) | 5.67 (0.82) | 2.33 (3.01) | 6.10 (0.68) | 3.33 (2.25) |
| Palate | 8 | 0.75 (2.12) | 4.38 (2.88) | 1.88 (2.80) | 1.88 (2.80) | 0.50 (1.41) | 3.38 (2.56) | 5.00 (2.27) | 2.12 (3.04) | 4.38 (2.77) | 1.25 (2.55) |
| RMT | 10 | 3.30 (2.50) | 4.5 (2.46) | 4.20 (3.05) | 3.95 (3.08) | 1.00 (2.11) | 4.50 (3.17) | 5.20 (1.87) | 2.10 (2.77) | 5.10 (1.91) | 2.50 (2.51) |
| Tongue | 98 | 2.32 (2.51) | 4.98 (2.43) | 3.62 (2.67) | 2.95 (2.59) | 1.72 (2.16) | 3.68 (2.64) | 5.06 (1.68) | 2.68 (2.56) | 5.79 (1.56) | 2.57 (2.75) |
| **HP Grade** |  |  |  |  |  |  |  |  |  |  |  |
| WDSCC | 166 | 2.01 (2.44) | 4.63 (2.44) | 3.82 (2.70) | 2.93 (2.63) | 1.81 (2.28) | 3.72 (2.66) | 5.18 (1.70) | 2.62 (2.49) | 5.75 (1.67) | 2.83 (2.64) |
| MDSCC | 106 | 2.57 (2.64) | 4.81 (2.48) | 3.32 (2.71) | 2.91 (2.72) | 1.24 (2.12) | 3.56 (2.62) | 5.10 (1.96) | 2.75 (2.61) | 5.80 (1.47) | 3.22 (2.85) |
| PDSCC | 10 | 4.53 (2.66) ^c^ | 5.20 (2.78) | 1.30 (2.16) ^d^ | 0.70 (1.64) ^e^ | 1.17 (2.04) | 4.54 (2.52) | 5.32 (0.44) | 1.10 (1.66) | 5.56 (2.04) | 2.90 (2.81) |
| **T Stage** |  |  |  |  |  |  |  |  |  |  |  |
| T_1_ & T_2_ | 77 | 2.32 (2.60) | 5.00 (2.54) | 2.89 (2.69) | 2.19 (2.45) | 1.28 (1.99) | 3.15 (2.69) | 4.8 (1.95) | 2.21 (2.45) | 5.65 (1.82) | 2.89 (2.74) |
| T_3_ & T_4_ | 205 | 2.31 (2.56) | 4.61 (2.43) ^f^ | 3.79 (2.70) ^g^ | 3.09 (2.70) ^g^ | 1.69 (2.30) | 3.90 (2.60) ^h^ | 5.29 (1.68) ^i^ | 2.77 (2.54) | 5.80 (1.52) | 3.01 (2.72) |
| **Node** |  |  |  |  |  |  |  |  |  |  |  |
| N- | 99 | 2.45 (2.62) | 4.72 (2.42) | 3.50 (2.85) | 2.66 (2.56) | 1.88 (2.31) | 3.74 (2.68) | 5.26 (1.62) | 2.86 (2.46) | 5.71 (1.61) | 2.74 (2.70) |
| N_+_ | 183 | 2.23 (2.54) | 4.71 (2.50) | 3.56 (2.66) | 2.94 (2.71) | 1.41 (2.16) | 3.67 (2.63) | 5.10 (1.85) | 2.49 (2.55) | 5.79 (1.61) | 3.11 (2.73) |
| **Clinical stage** |  |  |  |  |  |  |  |  |  |  |  |
| I & II | 33 | 2.60 (2.85) | 4.89 (2.48) | 2.75 (2.67) | 2.03 (2.53) | 1.02 (2.04) | 2.73 (2.46) | 4.61 (1.91) | 1.94 (2.42) | 5.85 (1.72) | 3.39 (2.78) |
| III & IV | 249 | 2.27 (2.53) | 4.69 (2.47) | 3.65 (2.72) | 2.95 (2.66) | 1.65 (2.24) ^j^ | 3.82 (2.64) ^k^ | 5.23 (1.74) | 2.71 (2.52) | 5.75 (1.59) | 2.92 (2.71) |

N/C ^a^p<0.01: Gender  ^b^p = 0.03; HP Grade ^c^p < 0.01; ^d^p = 0.01; ^e^p = 0.04; T Stage ^f^p = 0.02; ^g^p = 0.01; ^h^p = 0.03 ^i^p = 0.02; Clinical Stage ^j^p = 0.02; ^k^p = 0.01

Table S2. Immunohistochemical analysis of five biomarkers in normal oral tissues and OSCCs within Validation set

| **Clinical features** |  | **hnRNPK** | | **S100A7** | | **PTMA** | | **14-3-3σ** | | **14-3-3ζ** | |
| --- | --- | --- | --- | --- | --- | --- | --- | --- | --- | --- | --- |
|  | **N** | **Cytoplasmic** | **Nuclear** | **Cytoplasmic** | **Nuclear** | **Cytoplasmic** | **Nuclear** | **Cytoplasmic** | **Nuclear** | **Cytoplasmic** | **Nuclear** |
|  |  | **Mean (SD)** | **Mean (SD)** | **Mean (SD)** | **Mean (SD)** | **Mean (SD)** | **Mean(SD)** | **Mean (SD)** | **Mean (SD)** | **Mean (SD)** | **Mean (SD)** |
| **Normal** | 96 | 0.16 (0.69) | 6.03 (0.57) | 2.15 (1.89) | 1.87 (1.70) | 2.00 (1.37) | 4.60 (1.66) | 5.88 (0.44) | 2.90 (1.45) | 5.31 (1.24) | 2.25 (1.53) |
| **Cancer** | 135 | 0.16 (0.67) | 5.90 (0.70) | 3.67 (1.88) ^a^ | 3.24 (1.78) ^a^ | 2.33 (1.62) | 5.26 (1.30) ^a^ | 5.24 (1.56) ^a^ | 1.87 (1.81) ^a^ | 5.41 (0.96) | 1.35 (1.51) ^a^ |
| **Age (years)** |  |  |  |  |  |  |  |  |  |  |  |
| < 63 | 67 | 0.25 (0.84) | 5.91 (0.76) | 3.59 (1.99) | 3.11 (1.86) | 2.50 (1.54) | 5.18 (1.30) | 5.28 (1.58) | 2.19 (1.89) | 5.33 (1.04) | 1.48 (1.46) |
| ≥ 63 | 68 | 0.07 (0.43) | 5.88 (0.64) | 3.75 (1.77) | 3.37 (1.69) | 2.16 (1.69) | 5.33 (1.30) | 5.21 (1.56) | 1.55 (1.68) | 5.50 (0.87) | 1.23 (1.57) |
| **Gender** |  |  |  |  |  |  |  |  |  |  |  |
| Female | 52 | 0.04 (0.28) | 5.98 (0.66) | 3.36 (1.90) | 3.20 (1.78) | 1.87 (1.34) | 5.28 (1.33) | 5.21 (1.81) | 1.92 (1.99) | 5.58 (0.70) | 1.48 (1.69) |
| Male | 83 | 0.24 (0.82) | 5.84 (0.72) | 3.86 (1.84) | 3.27 (1.79) | 2.62 (1.72) ^b^ | 5.25 (1.29) | 5.26 (1.40) | 1.84 (1.70) | 5.31 (1.08) | 1.27 (1.40) |
| **Site** |  |  |  |  |  |  |  |  |  |  |  |
| Alveolus | 2 | 0.00 (0.00) | 5.83 (0.24) | 3.50 (0.71) | 2.00 (1.41) | 0.83 (1.18) | 5.57 (0.33) | 6.00 (0.00) | 1.00 (1.41) | 6.00 (0.00) | 1.50 (2.12) |
| BM | 14 | 0.00 (0.00) | 5.99 (0.76) | 3.25 (1.19) | 3.07 (1.49) | 2.00 (1.85) | 5.81 (0.89) | 4.52 (2.46) | 1.11 (1.71) | 5.62 (0.53) | 0.91 (1.14) |
| Mandible | 8 | 0.00 (0.00) | 5.95 (0.57) | 3.75 (1.16) | 3.88 (1.13) | 2.25 (1.58) | 5.63 (1.26) | 2.75 (2.46) | 0.75 (1.49) | 4.91 (1.31) | 1.30 (2.02) |
| Lip | 1 | 0.00 (-----) | 5.67 (-----) | 1.00 (-----) | 1.00 (-----) | 0.33 (-----) | 5.67 (-----) | 5.67 (-----) | 0.00 (-----) | 4.00 (-----) | 1.00 (-----) |
| Palate | 2 | 0.00 (0.00) | 6.50 (0.71) | 3.00 (1.41) | 3.00 (0.00) | 2.83 (1.65) | 6.17 (0.24) | 5.67 (0.47) | 0.00 (0.00) | 6.00 (0.00) | 0.00 (0.00) |
| RMT | --- | --- | --- | --- | --- | --- | --- | --- | --- | --- | --- |
| Tongue | 108 | 0.20 (0.75) | 5.87 (0.71) | 3.76 (2.00) | 3.26 (1.87) | 2.41 (1.60) | 5.13 (1.35) | 5.5 (1.14) | 2.12 (1.80) | 5.42 (0.97) | 1.44 (1.53) |
| **HP Grade** |  |  |  |  |  |  |  |  |  |  |  |
| WDSCC | 33 | 0.27 (0.76) | 5.96 (0.97) | 4.23 (1.78) | 3.50 (1.63) | 2.84 (1.44) | 5.25 (1.16) | 5.48 (1.22) | 1.92 (1.79) | 5.34 (1.25) | 0.94 (1.29) |
| MDSCC | 87 | 0.13 (0.66) | 5.89 (0.58) | 3.65 (1.82) | 3.33 (1.75) | 2.19 (1.68) | 5.18 (1.42) | 5.33 (1.52) | 1.98 (1.87) | 5.51 (0.83) | 1.69 (1.58) |
| PDSCC | 15 | 0.13 (0.52) | 5.78 (0.71) | 2.53 (1.96) ^c^ | 2.13 (2.00) | 2.00 (1.51) | 5.75 (0.65) | 4.21 (2.10) ^c^ | 1.13 (1.41) | 5.01 (0.80) ^d^ | 0.32 (0.76) ^e^ |
| **T Stage** |  |  |  |  |  |  |  |  |  |  |  |
| T_1_ & T_2_ | 102 | 0.15 (0.67) | 5.90 (0.74) | 3.74 (1.96) | 3.30 (1.83) | 2.34 (1.62) | 5.27 (1.26) | 5.43 (1.32) | 1.81 (1.79) | 5.33 (1.05) | 1.26 (1.50) |
| T_3_ & T_4_ | 33 | 0.21 (0.70) | 5.89 (0.58) | 3.45 (1.60) | 3.06 (1.62) | 2.30 (1.65) | 5.22 (1.42) | 4.67 (2.06) | 2.05 (1.88) | 5.67 (0.49) | 1.65 (1.55) |
| **Node** |  |  |  |  |  |  |  |  |  |  |  |
| N- | 76 | 0.14 (0.71) | 5.96 (0.66) | 3.81 (1.88) | 3.36 (1.79) | 2.52 (1.69) | 5.27 (1.33) | 5.34 (1.39) | 1.74 (1.85) | 5.42 (1.00) | 1.34 (1.42) |
| N_+_ | 59 | 0.19 (0.63) | 5.82 (0.75) | 3.49 (1.87) | 3.08 (1.77) | 2.08 (1.51) | 5.24 (1.27) | 5.12 (1.76) | 2.04 (1.76) | 5.41 (0.9) | 1.37 (1.64) |
| **Clinical stage** |  |  |  |  |  |  |  |  |  |  |  |
| I & II | 62 | 0.15 (0.74) | 5.94 (0.70) | 3.92 (1.88) | 3.41 (1.74) | 2.42 (1.62) | 5.31 (1.30) | 5.43 (1.33) | 1.64 (1.90) | 5.37 (1.08) | 1.31 (1.47) |
| III & IV | 73 | 0.18 (0.61) | 5.86 (0.70) | 3.46 (1.86) | 3.09 (1.81) | 2.25 (1.63) | 5.21 (1.30) | 5.08 (1.73) | 2.07 (1.72) | 5.45 (0.84) | 1.39 (1.56) |

N/C^a^p < 0.01; Gender  ^b^p = 0.02; HP Grade ^c^p = 0.02; ^d^p = 0.01; ^e^p = 0.04.

**Table S3. Univariable** Cox Regression analyses

|  | **Test set**  (n=282, events =122) | | **External validation**  (n = 135, events = 80) | |
| --- | --- | --- | --- | --- |
| **Predictors** | **HR [95% CI]** | **p** | **HR [95% CI]** | **p** |
| Nuclear S100A7 | 1.15 [1.08, 1.24] | < 0.001 | 0.99 [0.88, 1.11] | 0.86 |
| Cytoplasmic S100A7 | 1.12 [1.04, 1.2] | 0.002 | 0.95 [0.85, 1.05] | 0.33 |
| Nuclear hnRNPK | 1.14 [1.04, 1.24] | 0.003 | 0.79 [0.60, 1.04] | 0.09 |
| Cytoplasmic hnRNPK | 1.15 [1.08, 1.23] | < 0.001 | 1.35 [1.05, 1.74] | 0.02 |
| Nuclear PTMA | 1.19 [1.1, 1.27] | < 0.001 | 1.04 [0.86, 1.25] | 0.69 |
| Cytoplasmic PTMA | 0.96 [0.88, 1.05] | 0.039 | 0.80 [0.69, 0.93] | 0.003 |
| Nuclear 14-3-3σ | 0.96 [0.89, 1.03] | 0.24 | 0.72 [0.62, 0.83] | < 0.001 |
| Cytoplasmic 14-3-3σ | 1.15 [1.02, 1.29] | 0.02 | 0.83 [0.74, 0.94] | 0.002 |
| Nuclear 14-3-3**ζ** | 1.06 [0.99, 1.13] | 0.08 | 0.82 [0.70, 0.96] | 0.01 |
| Cytoplasmic 14-3-3**ζ** | 1.19 [1.02, 1.4] | 0.03 | 0.80 [0.67, 0.95] | 0.01 |

**Table S4. Multivariable** Cox Regression analyses

|  | **Test set**  (n=282, events =122) | | **Internal validation**  (9999 bootstrap samples) | | **External validation**  (n = 135, events = 80) | |
| --- | --- | --- | --- | --- | --- | --- |
| **Predictors** | **HR [95% CI]** | **p** | **HR [95% CI]** | **P** | **HR [95% CI]** | **p** |
| Nuclear S100A7 | 1.15 [1.07, 1.23] | < 0.001 | 1.15 [1.07, 1.24] | < 0.001 | 0.99 [0.88, 1.11] | 0.82 |
| Cytoplasmic hnRNPK | 1.16 [1.08, 1.24] | < 0.001 | 1.16 [1.08, 1.25] | < 0.001 | 1.58 [1.19, 2.09] | 0.001 |
| Nuclear PTMA | 1.23 [1.14, 1.33] | < 0.001 | 1.24 [1.14, 1.34] | < 0.001 | 1.09 [0.89, 1.34] | 0.38 |
| Cytoplasmic PTMA | 0.83 [0.76, 0.91] | < 0.001 | 0.83 [0.74, 0.91] | < 0.001 | 0.77 [0.67, 0.90] | < 0.001 |
| **Discriminatory value** | c-statistic = 0.69 | | c-statistic = 0.68 ^€^ | | c-statistic = 0.65 | |

^€^ Optimism-corrected index

Table S5: Interaction tests between biomarkers signature score and clinical parameters

|  | **Test set**  (n=282, events =122) | | **External validation**  (n = 135, events = 80) | |
| --- | --- | --- | --- | --- |
| **Clinical parameter** | **HR [95% CI]** | **p** | **HR [95% CI]** | **p** |
| Nodal status | 0.92 [0.86, 0.99] | 0.02 | 1.09 [0.97, 1.23] | 0.13 |
| Tumor stage | 0.99 [0.93, 1.05] | 0.76 | 1.12 [0.99, 1.28] | 0.08 |
| Clinical stage | 0.95 [0.86, 1.04] | 0.25 | 1.14 [1.01, 1.28] | 0.03 |
| Histology grade | 1.00 [0.95, 1.04] | 0.85 | 1.08 [0.97, 1.21] | 0.15 |

Figure Legend

**Figure S1. Distributions of biomarker scores in the Test and Validation sets**
