# Supplementary figures and images for: Prediction of recurrence-free survival using a protein expression-based risk classifier for head and neck cancer
Source: Oncogenesis. 2015 Apr 20;4(4):e147–. doi: 10.1038/oncsis.2015.7 (PMC4491610; doi:10.1038/oncsis.2015.7)

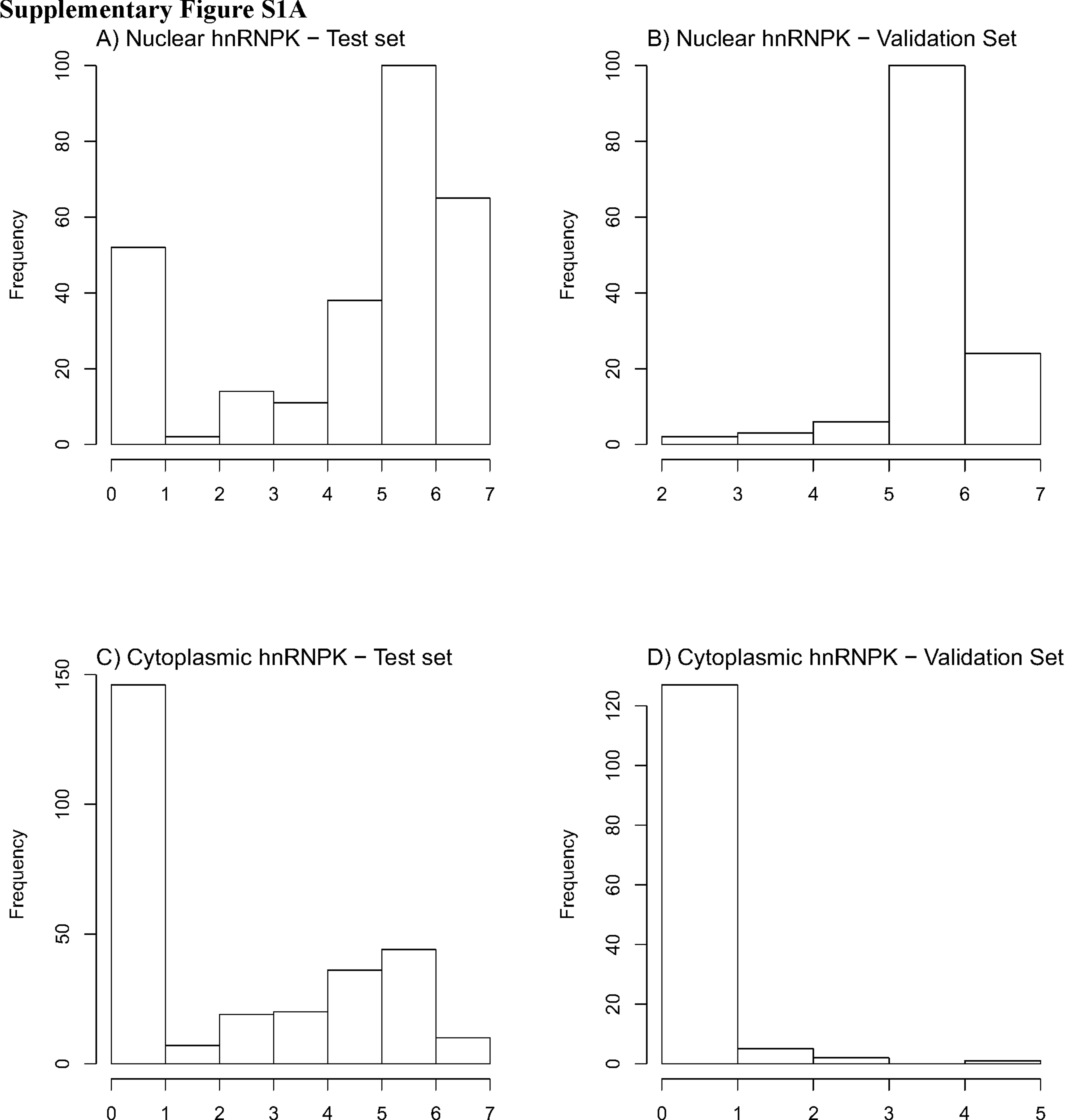

Supplement: Supplementary Figure S1A [file oncsis20157x2.tif]

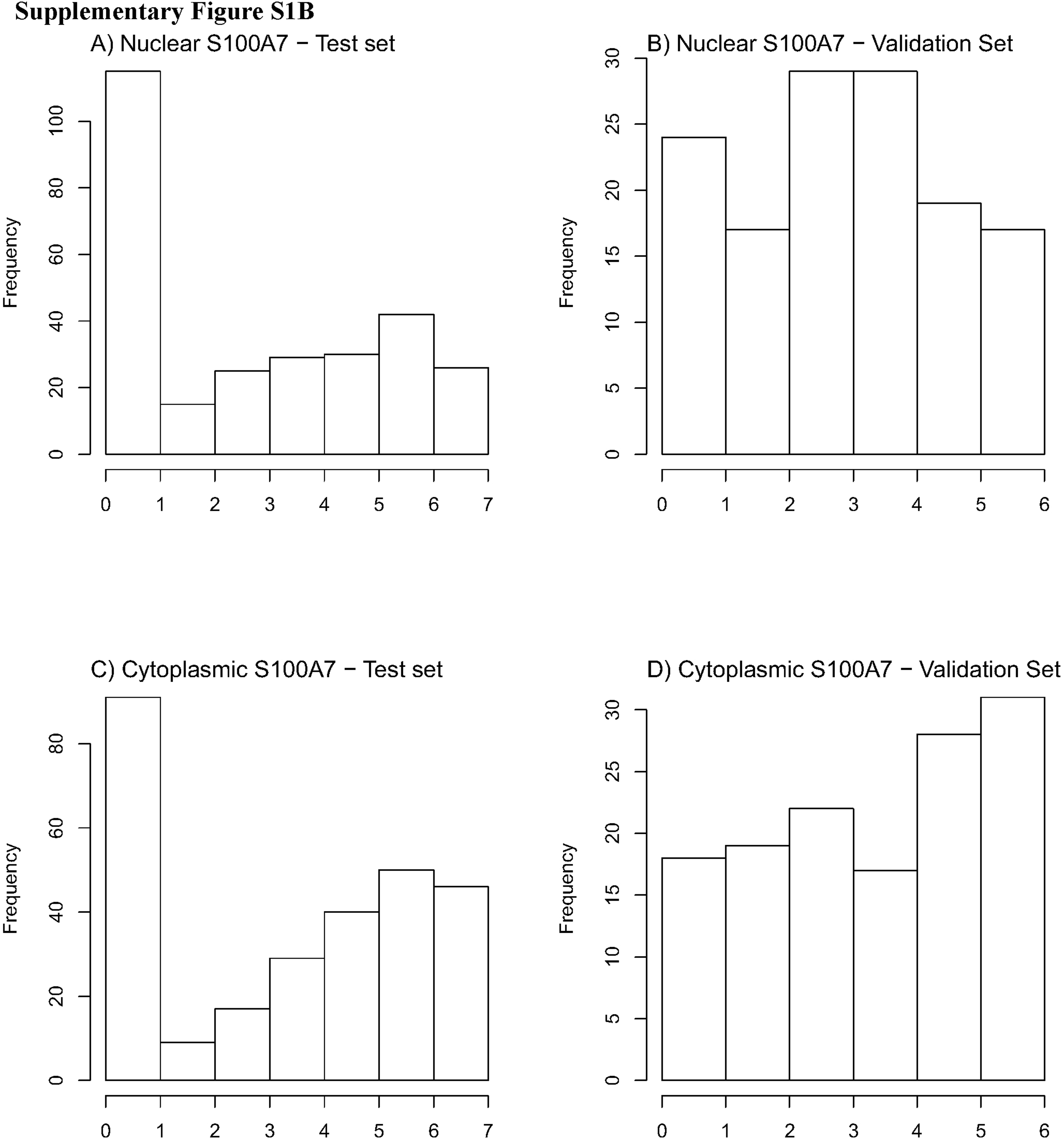

Supplement: Supplementary Figure S1B [file oncsis20157x3.tif]

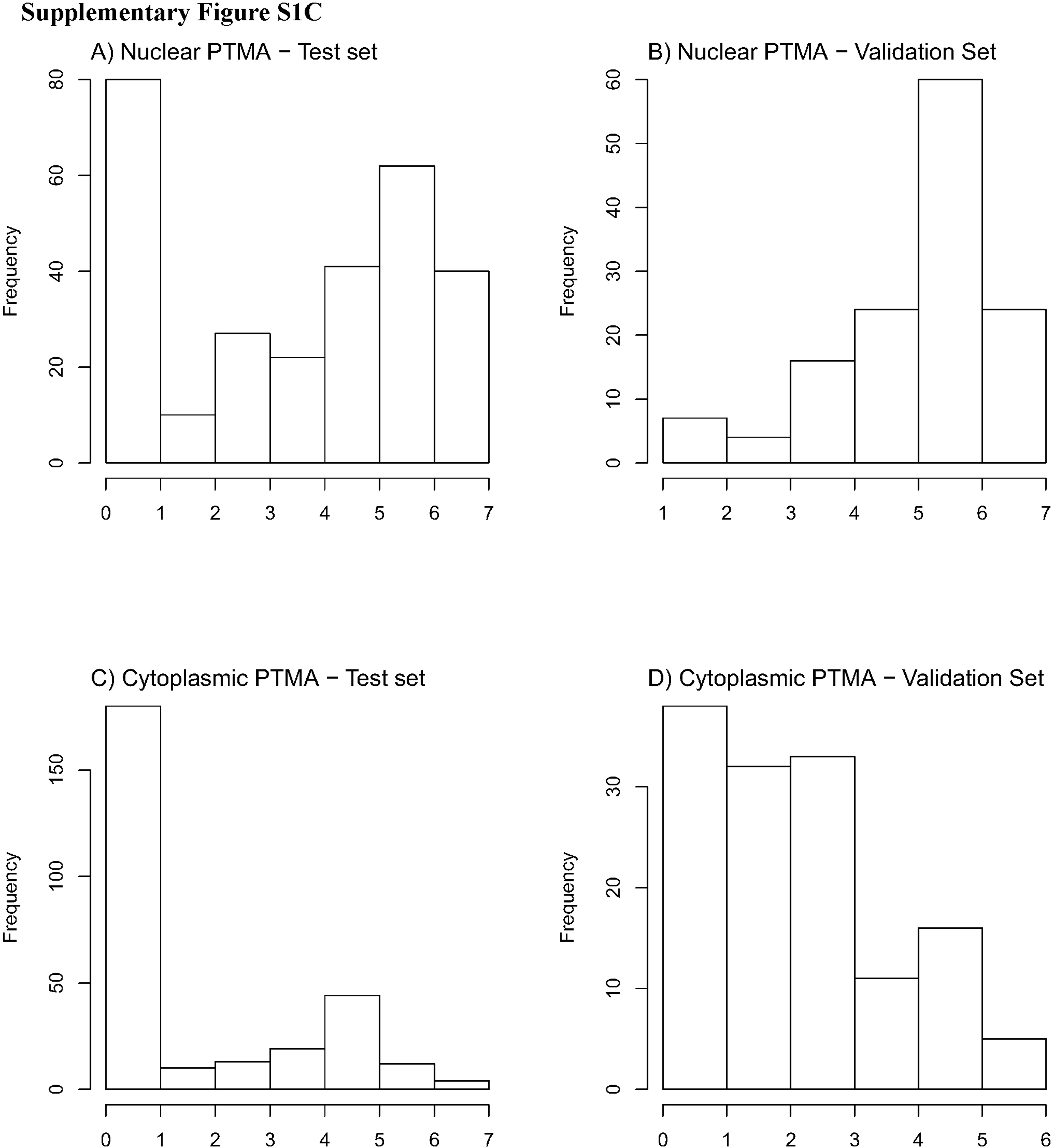

Supplement: Supplementary Figure S1C [file oncsis20157x4.tif]

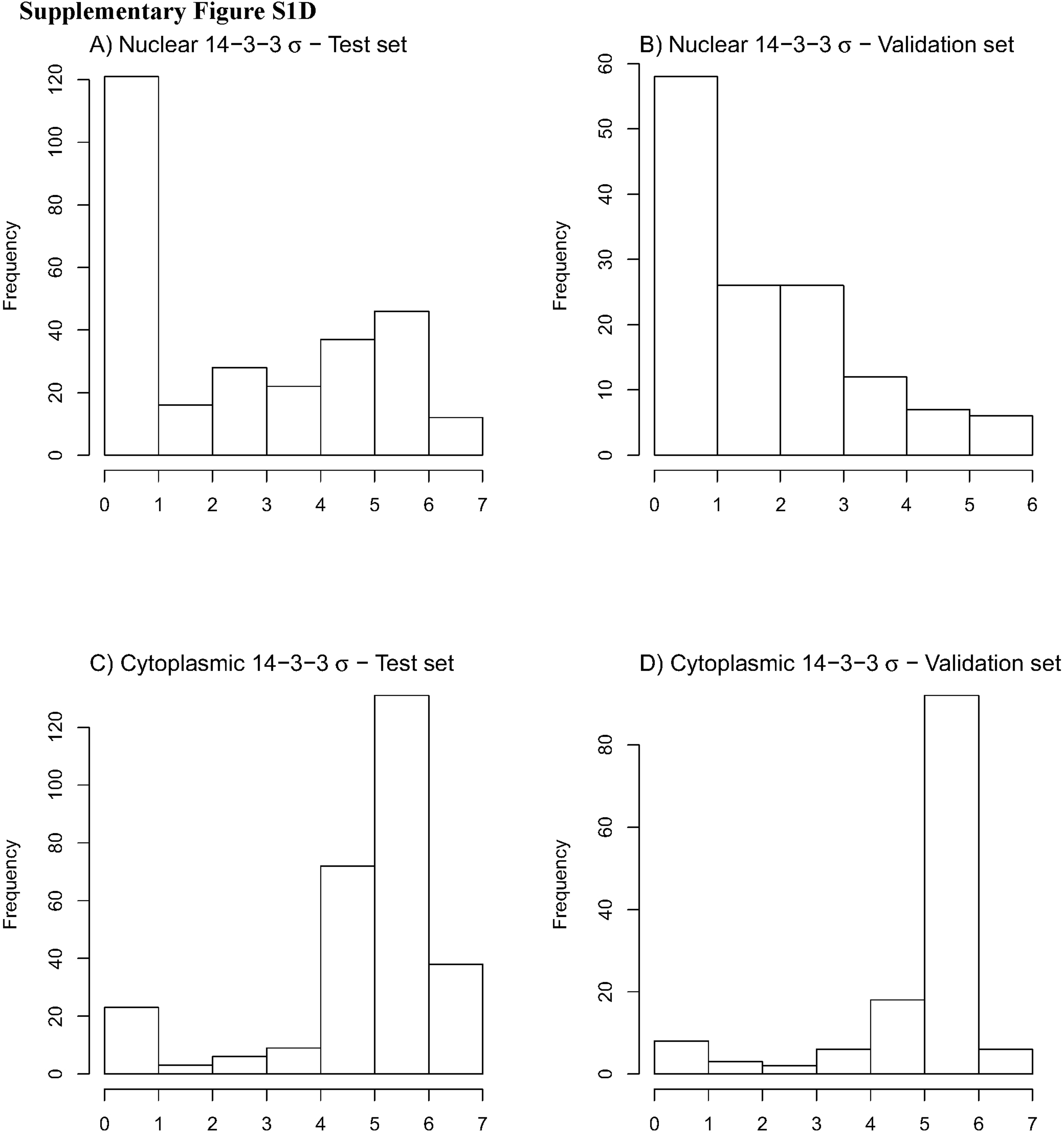

Supplement: Supplementary Figure S1D [file oncsis20157x5.tif]

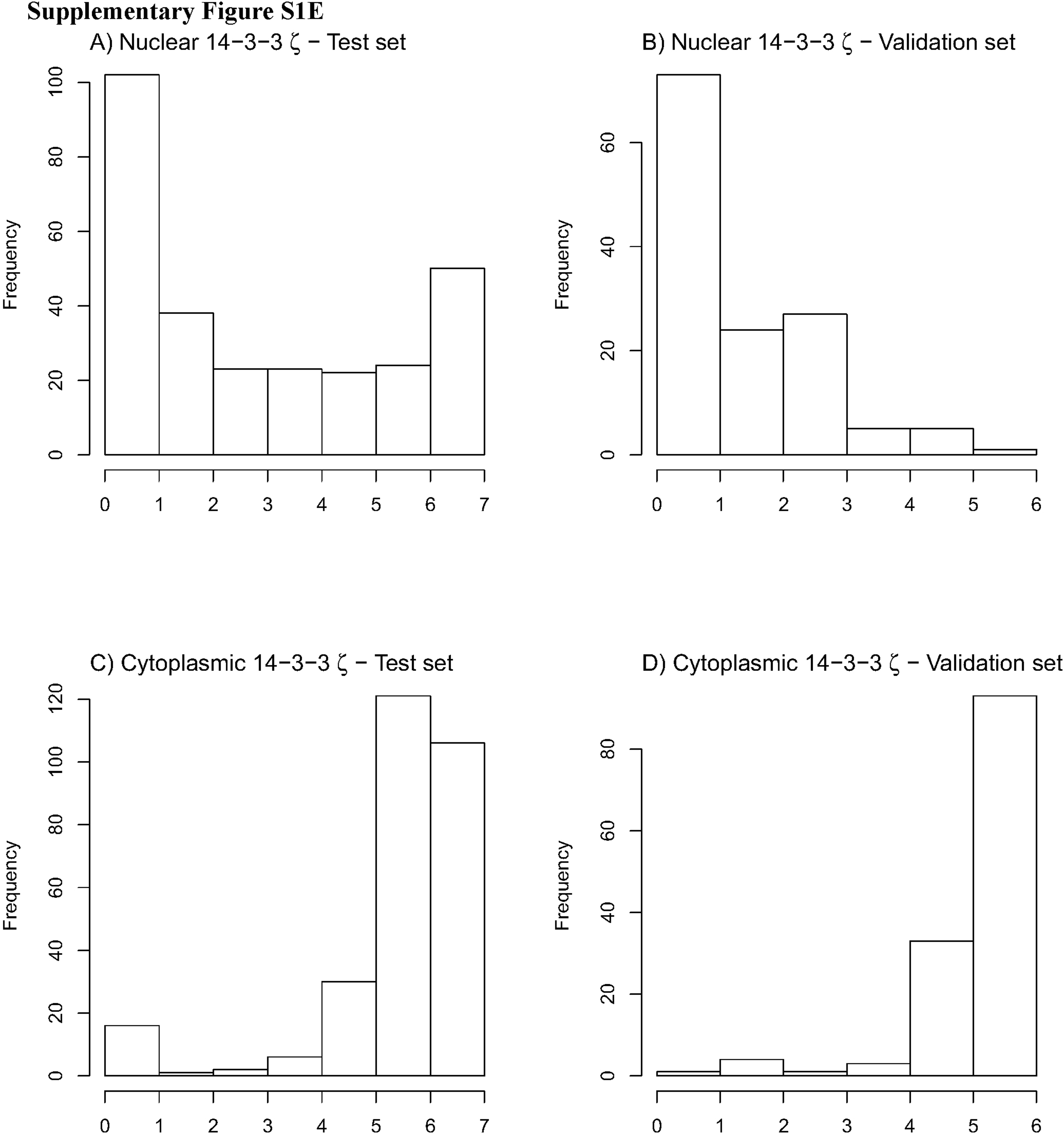

Supplement: Supplementary Figure S1E [file oncsis20157x6.tif]
